# A Triplet/Singlet Ground-State Switch via the Steric Inhibition of Conjugation in 4,6-Bis(trifluoromethyl)-1,3-phenylene Bisnitroxide

**DOI:** 10.3390/molecules29010070

**Published:** 2023-12-21

**Authors:** Nagito Haga, Takayuki Ishida

**Affiliations:** Department of Engineering Science, The University of Electro-Communications, Chofu 182-8585, Tokyo Prefecture, Japan

**Keywords:** aminoxyl, magnetic properties, biradical, exchange interaction, DFT calculation

## Abstract

Ground triplet 4,6-bis(trifluoromethyl)-1,3-phenylene bis(*tert*-butyl nitroxide) (TF2PBN) reacted with [Y(hfac)_3_(H_2_O)_2_] (hfac = 1,1,1,5,5,5-hexafluoropentane-2,4-dionate), affording a doubly hydrogen-bonded adduct [Y(hfac)_3_(H_2_O)_2_(TF2PBN)]. The biradical was recovered from the adduct through recrystallization. Crystallographic analysis indicates that the torsion angles (|*θ*| ≤ 90°) between the benzene ring and nitroxide groups were 74.9 and 84.8° in the adduct, which are larger than those of the starting material TF2PBN. Steric congestion due to *o*-trifluoromethyl groups gives rise to the reduction of π-conjugation. Two hydrogen bonds enhance this deformation. Susceptometry of the adduct indicates a ground singlet with 2*J*/*k*_B_ = −128(2) K, where 2*J* corresponds to the singlet–triplet gap. The observed magneto-structure relation is qualitatively consistent with Rajca’s pioneering work. A density functional theory calculation at the UB3LYP/6-311+G(2d,p) level using the atomic coordinates determined provided a result of 2*J*/*k*_B_ = −162.3 K for the adduct, whilst the corresponding calculation on intact TF2PBN provided +87.2 K. After a comparison among a few known compounds, the 2*J* vs. |*θ*| plot shows a negative slope with a critical torsion of 65(3)°. The ferro- and antiferromagnetic coupling contributions are balanced in TF2PBN, being responsible for ground-state interconversion by means of small structural perturbation like hydrogen bonds.

## 1. Introduction

Coordination compounds provide us a great opportunity for the development of spin-related functional materials [1,2,3,4,5]. Rational design, chemical modification, and physical modulation, etc. have attracted great attention to magnetic materials controlled by external stimuli [6,7]. To apply ground triplet biradicals and ground high-spin oligoradicals to materials chemistry [8,9,10,11,12,13,14,15,16], the choice of radical species is crucial. It has been theoretically and experimentally clarified that 1,3-phenylene-bridges serve as a robust ferromagnetic coupler in the context of the spin-polarization mechanism or topological rule [4,5,11,12,13,14,15,16,17,18,19]. Triphenylmethyls and diphenylcarbenes are the most popular paramagnetic centers in this research field, but heteroatomic ones can also be utilized as a spin source [17,18,19,20,21,22]. Nitroxides or aminoxyls are persistent radicals and are available for various aspects in chemistry, physics, and biology [20,21,22,23] thanks to the stability achieved through the resonance of the following canonical formulas: R^1^–N(–O^•^)–R^2^←→R^1^–N^+•^(–O^–^)–R^2^. The non-bonding molecular orbital (NBMO) belongs to a π* symmetry and has comparable contributions from N and O atomic orbitals. The nitroxide oxygen atom can participate in hydrogen (H) bonds [24] as well as coordination bonds [1,2,3,4,5] and is reasonably understood from the latter canonical formula.

**Scheme 1:**
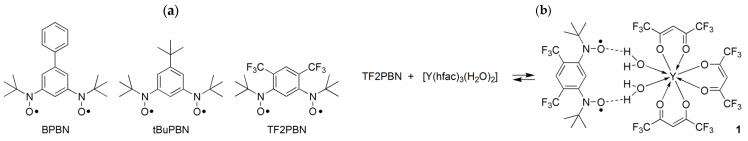
(**a**) Structural formulas of BPBN, tBuPBN, and TF2PBN. (**b**) Reaction Scheme for [Y(hfac)_3_(H_2_O)_2_(TF2PBN)] (**1**).

There are two typical ways to stabilize organic radicals [25,26]. Bulky substituents will raise the energy level of a transition state and decrease the reaction rate, which is comprehended in terms of steric protection or kinetic persistency. π-Conjugated substituents will suppress the energy level of a reactant due to spin delocalization, often called the resonance effect or thermodynamic stability. Aromatic substituents usually have both contributions. The biphenyl-3,5-diyl bis(*tert*-butyl nitroxide) (BPBN) biradical and several related derivatives [27,28,29] (Scheme 1a) have enough persistency to be isolated under ambient conditions. They seem to be promising for the development of 2p, 2p-3d, and 2p-4f homo-/heterospin magnets. However, decomposition reactions of BPBNs have also been reported during further chemical reactions [30,31,32]. As for bulky aliphatic groups, 5-*tert*-butyl-1,3-phenylene bis(*tert*-butyl nitroxide), tBuPBN [33] (Scheme 1a), appears to be another attractive candidate with improved persistency and has actually been applied in coordination chemistry [34,35].

The sterically protecting groups of BPBN and tBuPBN, peripheral aryl and *tert*-butyl substituents, are located at the *m*-position with respect to the nitroxide groups. On the other hand, 4,6-bis(trifluoromethyl)-1,3-phenylene bis(*tert*-butyl nitroxide) (TF2PBN, Scheme 1a), originally synthesized by Rajca et al. [36], takes advantage of the *o*- and *p*-positions. This structural feature will enhance effective protection and, accordingly, persistency. In fact, it can be isolated and manipulated under ambient aerobic conditions. Furthermore, TF2PBN was reported to have triplet spin multiplicity at the ground state [36,37]. These characters seem to be suitable for magnetism-based materials chemistry. A methyl group at the *o*- or *p*-position is reported to destabilize phenyl nitroxides owing to a hydrogen abstraction producing quinoid compounds [32]. Thus, the roles of methyl and trifluoromethyl groups are strikingly different from each other in nitroxide chemistry.

We found that TF2PBN reacted with [Y(hfac)_3_(H_2_O)_2_] (hfac = 1,1,1,5,5,5-hexafluoropentane-2,4-dionate) [38] to produce a supramolecular discrete adduct, [Y(hfac)_3_(H_2_O)_2_(TF2PBN)] (abbreviated as **1** hereafter; Scheme 1b). The TF2PBN and [Y(hfac)_3_(H_2_O)_2_] portions are connected only with double H bonds and not coordination bonds. There is a 12-membered ring which seems to be rather rare in chelate chemistry, to our knowledge. Since Y^3+^ is diamagnetic, the present study is attributed to a research on genuine organic magnetism. Rajca and co-workers have already reported [Mn(hfac)_2_(TF2PBN)_2_] coordination compounds [37]. The nitroxide oxygen atoms are directly coordinated to the Mn^2+^ center, giving rise to considerable 2p-3d exchange coupling. Interestingly, the *m*-phenylene bridge was assumed to play the role of an antiferromagnetic coupler. In comparison with Rajca’s work, the present compound has an advantage in the unequivocal analysis of the exchange interaction in a ligand. In this report, we attempt to construct a framework for predicting *J* from the molecular structure and tuning *J* by structural deformation after conducting detailed structural and magnetic analyses of **1** together with related compounds.

## 2. Materials and Methods

The following procedure for the preparation of **1** is typical. After [Y(hfac)_3_(H_2_O)_2_] [38] (74.9 mg; 0.104 mmol) was dissolved in heptane (10 mL) at 90 °C, the resultant solution was combined with a dichloromethane solution (1 mL) containing TF2PBN [37] (38.4 mg; 0.0995 mmol) at room temperature. After 5 days, orange polycrystals of **1** and unchanged colorless [Y(hfac)_3_(H_2_O)_2_] were both precipitated; they were collected on a filter, washed, and air-dried. The total mass was 17.0 mg. IR (neat, attenuated total reflection) 3473, 3260, 3151, 2990, 1649, 1619, 1566, 1539, 1474, 1328, 1253, 1226, 1204, 1138, 1100, 1062, 953, 919, 808, 743, 662 cm^−1^. Anal. Calcd. for C_47.5_H_34.7_N_2_O_18.8_F_43.8_Y_2.1_ ([Y(hfac)_3_(H_2_O)_2_(TF2PBN)]· [Y(hfac)_3_(H_2_O)_2_]_1.1_: C, 29.21; H, 1.79; N, 1.43%. Found: C, 28.35; H, 1.56; N, 1.98%. On the basis of the elemental and magnetic analysis, the yield of **1** was estimated to be 8.8%. M.p. 123 °C (decomp.).

The above orange product was recrystallized from ethanol. Recovered red crystals were determined to be identical to the starting material TF2PBN, as confirmed by means of IR spectroscopy and a single-crystal X-ray diffraction analysis.

X-ray diffraction data of a single crystal of **1** and [Y(hfac)_3_(H_2_O)_2_] recovered from the above reaction were recorded on a Rigaku XtaLAB Synergy R HyPix diffractometer with graphite monochromated Mo Kα radiation (*λ* = 0.71073 Å) at 100 K. The *hkl* and intensity data were extracted using CrysAlisPro [39]. The structure was solved directly and expanded using Fourier techniques in the Olex2 program [40]. Hydrogen atoms were located at calculated positions. The parameters were refined using Shelxl [41]. Disorder models were applied to three CF_3_ groups in the [Y(hfac)_3_(H_2_O)_2_] moiety in **1** and to all CF_3_ groups in [Y(hfac)_3_(H_2_O)_2_]. The crystal data of **1** are as follows: C_31_H_27_N_2_O_10_F_24_Y, triclinic, *P*$\bar{1}$, *a* = 11.8139(2), *b* = 11.8703(2), *c* = 16.8620(3) Å, *α* = 79.8250(10), *β* = 84.9680(10), *γ* = 66.348(2)**°**, *V* = 2131.57(7) Å^3^, *Z* = 2, *d*_calc_ = 1.764 g cm^−3^, *μ*(Mo Kα) = 1.527 mm^−1^, *R*_int_ = 0.0201, *R*_1_(*I* > 2*σ*(*I*)) = 0.0295, *R*_w_(all data) = 0.0804, and GOF = 1.040 at 99.9(3) K with 11,851 independent reflections. Selected bond distances and angles are listed in Table 1. The crystal data of [Y(hfac)_3_(H_2_O)_2_] are as follows: C_15_H_7_F_18_O_8_Y, triclinic, *P*$\bar{1}$, *a* = 9.9136(4), *b* = 11.5271(4), *c* = 12.3329(4) Å, *α* = 67.952(3), *β* = 73.764(3), *γ* = 75.973(3)**°**, *V* = 1239.13(9) Å^3^, *Z* = 2, *d*_calc_ = 2.000 g cm^−3^, *μ*(Mo Kα) = 2.528 mm^−1^, *R*_int_ = 0.0179, *R*_1_(*I* > 2*σ*(*I*)) = 0.0299, *R*_w_(all data) = 0.0720, and GOF = 1.031 at 100.0(1) K with 6556 independent reflections. The experimental details and full geometrical parameter tables can be obtained using the CCDC reference numbers 2301482 and 2303226 for **1** and [Y(hfac)_3_(H_2_O)_2_], respectively. Powder X-ray diffraction (PXRD) data were recorded on a Rigaku SmartLab diffractometer using Cu K*α* radiation (*λ* = 1.54178 Å) at room temperature.

The magnetic susceptibilities of TF2PBN and **1** were measured on a Quantum Design MPMS3 SQUID magnetometer. The data were acquired at 0.5 T in a temperature range from 1.8 to 300 K. The magnetic responses were corrected with diamagnetic blank data of a gelatin capsule sample holder measured separately, and a further diamagnetic contribution was estimated from Pascal’s constants [42].

Density functional theory (DFT) calculations on TF2PBN, **1**, and related known compounds MO2PBN, BrMO2PBN, and MesBN (see below) were carried out using Gaussian16 Revision C.01 [43]. For molecular structures, see the Results section. The broken symmetry method [44,45,46] and the unrestricted B3LYP theory were applied to the basis sets of lanl2dz for Y and 6-311+G(2d,p) for other elements. Self-consistent field energies were calculated using the experimentally determined coordinates. In the spin Hamiltonian provided as Equation (1), the exchange coupling parameter was reduced using Yamaguchi’s equation (Equation (2)) [47,48].

(1) $$\hat{H}=-2J{\hat{S}}_{1}\cdot {\hat{S}}_{2}$$

(2) $$J=\frac{E_{BS}^{LS}-E^{HS}}{{\;\;\langle {\hat{S}}^{2}\rangle }^{HS}-{\langle {\hat{S}}^{2}\rangle }_{BS}^{LS}\;\;\;}$$

## 3. Results

### 3.1. Synthesis and Structural Analysis

The adduct, [Y(hfac)_3_(H_2_O)_2_(TF2PBN)] (**1**), was obtained by simply mixing the two starting materials. The product is completely stable below at least the melting point (123 °C (decomp.)) under ambient conditions. It should also be noted that no degradation was observed, unlike the BPBN case [30,31]. An IR absorption appeared around 3260 cm^−1^ after adduct formation, which can be assigned to H-bonded O-H stretching [49]. The reaction product was afforded as a mixture of **1** and the starting material, [Y(hfac)_3_(H_2_O)_2_], with a molar ratio 1/1.1, as evidenced by the elemental and IR spectroscopic analyses as well as the powder X-ray diffraction study (see below). The impurity, [Y(hfac)_3_(H_2_O)_2_], was present as a mixture at a crystalline level. Biradical TF2PBN can be recovered from **1** via recrystallization from ethanol. This finding indicates that not only are the present H bonds weak but also that the ground-state interconversion is reversible.

**Figure 1:**
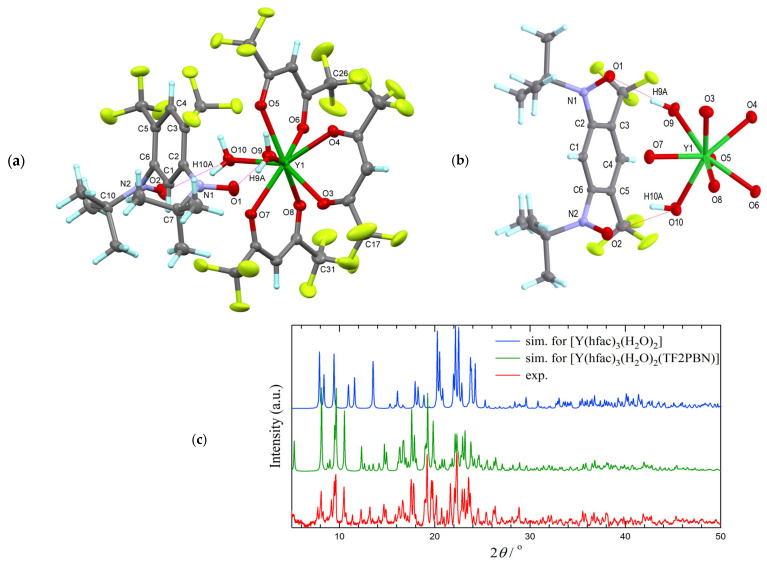
(**a**) Crystal structure of **1** with thermal ellipsoids at the 50% probability level. Major conformations are drawn for disordered CF_3_ groups at C17 (occupancy 0.640(4)), C26 (0.770(5)), and C31 (0.68(2)). Dotted lines stand for H bonds, denoted between the oxygen atoms. Selected atomic numbering is also shown. Atomic color codes: C, gray; H, turquoise; N, blue; O, red; F, yellow; Y, green. (**b**) An important portion after the C, H, and F atoms in hfac is omitted for the sake of clarity. (**c**) Simulated PXRD profiles for **1** and [Y(hfac)_3_(H_2_O)_2_] and experimental PXRD data for the product, **1**·[Y(hfac)_3_(H_2_O)_2_]_1.1_.

Figure 1a,b display the X-ray crystal structure of **1**, and experimental and simulated powder X-ray diffraction results are also shown in Figure 1c. No lattice solvent molecules are involved. The positions of three hfac groups cannot be symmetrically arranged and, accordingly, the two nitroxide groups are unequal. Double H bonds can be found in O1⋯H9A–O9 and O2⋯H10A–O10. The O1⋯O9 and O2⋯O10 distances are 2.70552(6) and 2.72070(5) Å, respectively. The N1–O1⋯O9 and N2–O2⋯O10 angles are 113.1067(13) and 123.2740(12)°, respectively, and the O1⋯O9–Y1 and O2⋯O10–Y1 angles are 141.6666(8) and 136.1056(13)°, respectively, being consistent with the H bond angles. The adduct molecule is discrete and magnetically isolated because the peripheral trifluoromethyl and *tert*-butyl groups bring about only a weak van der Waals interaction.

The N1–O1 and N2–O2 bond lengths in **1** are 1.2842(16) and 1.2850(16) Å, respectively, typical of nitroxide radicals [50]. Now, we compare the geometries between the intact TF2PBN molecule [36] and the TF2PBN moiety in **1** (Table 1). The geometrical parameters of the TF2PBN portion in [Mn(hfac)_2_(TF2PBN)_2_] [37] are also included in Table 1. All the geometrical parameters are quite similar to each other except for the torsion angles along O–N–Cα_(Ar)_–Cβ_(Ar)_ and the intramolecular O1⋯O2 distance. The torsion angles of TF2BN in **1** are closer to the right angle than those of the intact TF2PBN and the TF2PBN moiety in [Mn(hfac)_2_(TF2PBN)_2_], and the O1⋯O2 distance in **1** is the shortest. This finding indicates that the nitroxide oxygen atoms in **1** are the most dislocated from the benzene plane. As a consequence, the π-conjugation in **1** is the most severely inhibited between the benzene ring and nitroxide groups.

**Figure 2:**
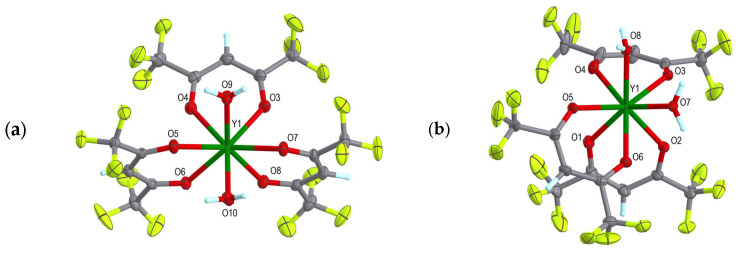
Crystal structure of (**a**) the [Y(hfac)_3_(H_2_O)_2_] moiety in **1** and (**b**) [Y(hfac)_3_(H_2_O)_2_] with thermal ellipsoids at the 50% probability level. Major conformations are drawn for disordered CF_3_ groups. Selected atomic numbering is also shown. For the atomic color codes, see Figure 1.

The Y^3+^ ion in **1** was eight-coordinate, and the coordination structure is best described as a square antiprism (SAPR) (Figure 2a). The SHAPE analysis [51] indicates that the continuous shape measure was 0.485 with respect to an ideal SAPR-8 reference. The H-donor OH groups in two aqua ligands (O9 and O10) are arranged in a *trans* position in a basal square. The O9–Y1–O10 angle is 104.79(4)°, and the interatomic distance between O9 and O10 is 3.73555(7) Å in **1**. The X-ray crystal structure of [Y(hfac)_3_(H_2_O)_2_] was also determined (Figure 2b), and those of lanthanide analogs [Ln(hfac)_3_(H_2_O)_2_] (Ln = Gd [CCDC 2300883], Ho [52] and Er [53]) are known. They are isomorphous, which can be reasonably understood from the ionic radii (1.05, 1.02, and 1.00 Å, respectively, vs. 1.02 Å for Y [54]). Two aqua ligands are always located in a *cis* position of a basal plane of the SAPR. The O_aq_–RE–O_aq_ angle is ca. 70.7–70.8°, with an O_aq_⋯O_aq_ distance of ca. 2.69–2.77 Å. Such structural flexibility seems to be beneficial to accommodate the strain accompanied by the 12-membered ring formation.

**Scheme 2:**
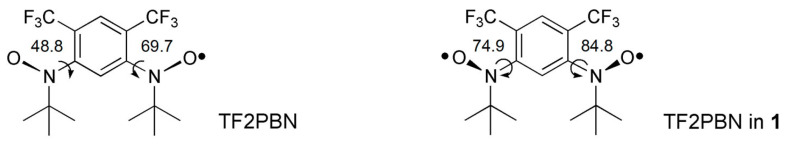
The torsion angles (**left**) before and (**right**) after adduct formation. *θ*_1_ and *θ*_2_ are shown.

Interestingly, intact TF2PBN already has a disrotatory conformation of the two nitroxide groups, leading to an approximate *C*_S_ symmetry and not *C*_2_ symmetry, as indicated by the positive and negative torsion angles (Scheme 2, left). Here, the torsion angle *θ* was chosen as |*θ*| ≤ 90°. After the association reaction, the O1⋯O2 distance in TF2PBN became shorter (Scheme 2, right), while the O_aq_⋯O_aq_ distance in [Y(hfac)_3_(H_2_O)_2_] became wider (6.339(16) vs. 5.8868(1) Å, Table 1) thanks to an attractive H-bonding interaction. This motion brings about the larger torsion around the O–N–Cα_(Ar)_–Cβ_(Ar)_.

### 3.2. Magnetic Analysis

**Figure 3:**
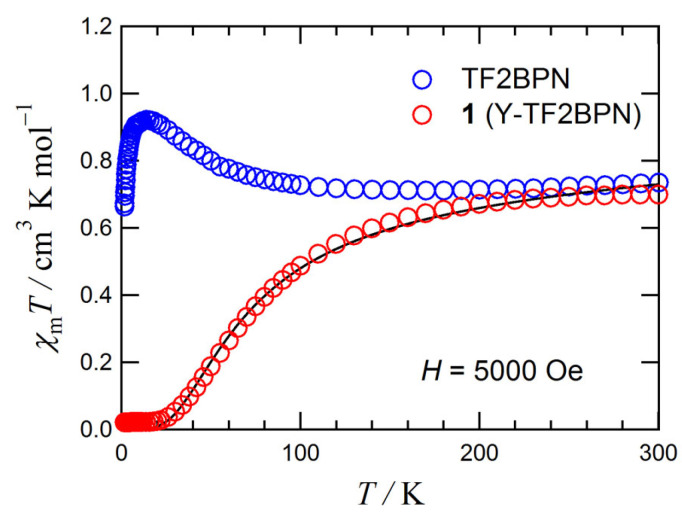
The *χ*_m_*T* vs. *T* plot for TF2PBN and **1**. The solid line represents the theoretical fit to the data on **1**. For the equation and parameters, see the text.

Figure 3 shows the magnetic susceptibility results for the starting material TF2PBN and **1**·[Y(hfac)_3_(H_2_O)_2_]_1.1_. The diamagnetic impurity at a polycrystalline level does not disturb the magnetic analysis, so the plot can practically be regarded as that of **1**. The *χ*_m_*T* value of **1** shows a monotonic decrease upon cooling, indicating the presence of antiferromagnetic coupling. Finally, the *χ*_m_*T* value was practically null at the base temperature (1.8 K), indicating the high purity of the biradical species as a paramagnetic source. If unreacted or overreacted species were present, the *χ*_m_*T* bias would appear as an *S* = 1/2 Curie impurity.

In sharp contrast, the ground sate of TF2PBN has been reported to be triplet with 2*J*/*k*_B_ = 40–80 K [36,37], which we were able to confirm independently (Figure 3). The *χ*_m_*T* value of TF2PBN shows an increase upon cooling, indicating the presence of ferromagnetic coupling. The final drop may originate in an intermolecular interaction.

The data of **1** were analyzed according to the Bleaney-Bowers formula (Equation (3)) [55]. Here, *χ*_m_ is the molar magnetic susceptibility, *T* is the absolute temperature, *J* is the exchange coupling constant, *g* is the Landé *g* factor, *N*_A_ is Avogadro’s number, *μ*_B_ is the Bohr magneton, and *k*_B_ is the Boltzmann constant. The singlet–triplet energy gap corresponds to 2*J*. The best optimized parameter was 2*J*/*k*_B_ = −128(2) K with the *g* value fixed to 2.006, a typical value for nitroxides [10]. The calculation curve almost reproduces the experimental data.

(3) $$\chi_{m}=\frac{2N_{A}g^{2}\mu_{B}^{2}}{k_{B}T}\;\frac{1}{3+exp(-2J/k_{B})}$$

There seem to be two possible radical–radical exchange pathways. One is through the *m*-phenylene bridge, and the other occurs through the diamagnetic Y^3+^ ion, namely, a superexchange mechanism across (N)O-H-O-Y-O-H-O(N). As shown in the plot for **1**, much stronger exchange coupling is observed than expected from the superexchange mechanism, which is typically of the order of 10 to 20 K [56,57]. The observed antiferromagnetic exchange coupling must be ascribed to the *m*-phenylene bridge.

There have been several reports on rule-breaking compounds regarding the ground states anticipated from Kekulé or non-Kekulé structural formulas [58,59,60,61]. Rajca et al. reported a pioneering work in which antiferromagnetic coupling was assigned to the *m*-phenylene-bridge in TF2PBN-Mn^II^(hfac)_2_-TF2PBN according to the approximate spin model (*S* = 1/2)–(*S* = 3/2)–(*S* = 1/2) [37]. In comparison to the Mn complex, compound **1** carries only two radical spins with only slight perturbations, leading to a highly reliable evaluation of *J*. To confirm these assumptions and construct a possible magneto-structure relationship, we performed calculations.

### 3.3. DFT Calculation Analysis

**Figure 4:**
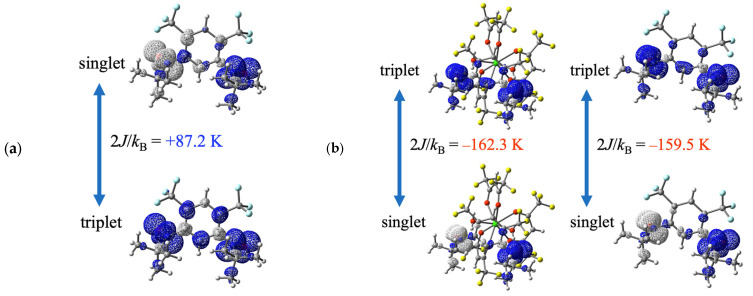
Relative energy levels of triplet and broken-symmetry singlet states, calculated at the UB3LYP/6-311+G(2d,p) level. Spin density surfaces are drawn at the 0.002 e^−^ Å^−3^ level with blue and white lobes for the positive and negative spin densities, respectively. (**a**) The self-consistent field (SCF) energies of TF2PBN are −1480.8374662573 au with <*S*^2^> = 2.0114 and −1480.8373276154 au with <*S*^2^> = 1.0080 for the triplet and singlet states, respectively. (**b**) (**Left**) The SCF energies of **1** are −4494.9085419557 au with <*S*^2^> = 2.0094 and −4494.9088003966 au with <*S*^2^> = 1.0041 for the triplet and singlet states, respectively. (**Right**) The SCF energies of the TF2PBN portion in **1** are −1480.9064795589 au with <*S*^2^> = 2.0094 and −1480.9067334369 au with <*S*^2^> = 1.0045 for the triplet and singlet states, respectively. Ferro- and antiferromagnetic *J* values are marked in blue and red, respectively. For the atomic color codes, see Figure 1.

A density functional theory (DFT) calculation on the UB3LYP/6-311+G(2d,p) level using the geometry determined for TF2PBN [36] afforded a triplet ground state with 2*J*/*k*_B_ = +87.2 K (Figure 4a). The intramolecular coupling was semi-quantitatively supported by the theoretical calculation. It is quite normal that *m*-phenylene bis(nitroxides) possesses a ground triplet state [62,63,64,65]. On the other hand, the corresponding calculation for **1** provided an antiferromagnetic interaction with 2*J*/*k*_B_ = −162.3 K (Figure 4b, left). To elucidate the presence or absence of superexchange coupling through the H-O-Y-O-H bridge, we applied the same calculation protocol to the virtual molecule TF2PBN after the [Y(hfac)_3_(H_2_O)_2_] portion was removed from **1** (Figure 4b, right). Antiferromagnetic coupling was again calculated to be 2*J*/*k*_B_ = −159.5 K. The two comparable 2*J* values tell us that the antiferromagnetic *m*-phenylene pathway is plausible. *m*-Phenylene bis(nitroxides) with a ground singlet state seem to be rather rare [37,66,67,68].

## 4. Discussion

Figure 4a indicates the spin distribution on the *m*-phenylene ring, while Figure 4b shows a much smaller spin density there. This picture clarifies that the spin polarization scheme hardly works in **1**. The *o*-trifluoromethyl group has interference against the adjacent *tert*-butyl nitroxide group, giving rise to the steric inhibition of π-conjugation. Instead, in the out-of-plane deformed structure, a through-space interaction seems to be operative [66,69] because σ-type bonding and antibonding contributions appeared in the benzene bridge and a possible interaction produced a *syn* conformation. According to the ab initio study, the antiferromagnetic couplings are due to a through-bond interaction which increases the energy difference between the symmetric and antisymmetric NBMOs [70].

The present result is consistent with the previous work on the post-Hartree-Fock CAS calculation of TF2PBN [71,72]. The exchange coupling values are plotted in the matrix of two torsion angles, *θ*_1_ and *θ*_2_, and almost four basins showing antiferromagnetic or very small coupling appeared regarding the severe out-of-plane conformation. The present values (Scheme 2), *θ*_1_ = −48.8° and *θ*_2_ = 69.7° for the starting material TF2PBN and *θ*_1_ = −74.9° and *θ*_2_ = 84.8° for complex **1**, belong to the ferro- and antiferromagnetic regions, respectively. Therefore, both post-Hartree-Fock CAS [71,72] and DFT (this work) calculations reproduced the experimental results well. This finding seems to be very helpful for researchers who do not specialize in calculation because a low-cost DFT treatment could afford a reliable output.

**Scheme 3:**
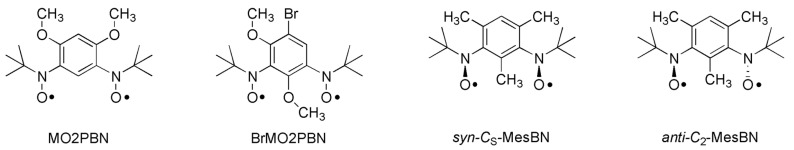
Structural formulas of MO2PBN, BrMO2PBN, *syn-C*_S_-MesBN, and *anti-C*_2_-MesBN.

**Figure 5:**
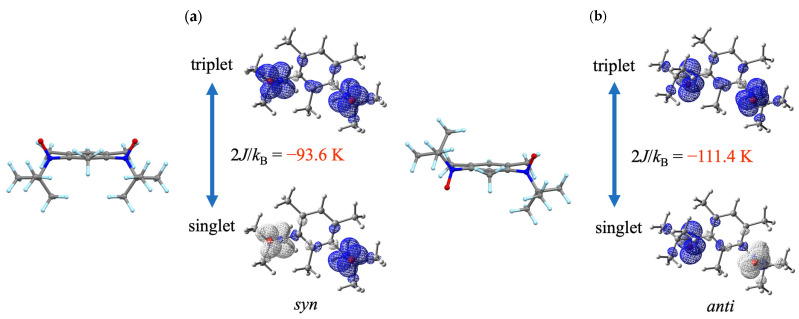
Relative energy levels of triplet and broken-symmetry singlet states, calculated at the UB3LYP/6-311+G(2d,p)//UB3LYP/6-31G(d) level. Optimized geometries were also shown. (**a**) The SCF energies of *syn*-MesBN are −924.7811231198 au with <*S*^2^> = 2.0095 and −924.7812718628 au with <*S*^2^> = 1.0060 for the triplet and singlet states, respectively. (**b**) The SCF energies of *anti*-MesBN are −924.7809370560 au with <*S*^2^> = 2.0096 and −924.7811143077 au with <*S*^2^> = 1.0056 for the triplet and singlet states, respectively. Spin density surfaces are drawn at the 0.002 e^−^ Å^−3^ level with blue and white lobes for the positive and negative spin densities, respectively. Antiferromagnetic *J* values are marked in red. For the atomic color codes, see Figure 1.

Iwamura et al. reported that biradical 4,6-dimethoxy-1,3-phenylene bis(*tert*-butyl nitroxide) (abbreviated as MO2PBN, Scheme 3) has *θ*_1_ = −75.3 and *θ*_2_ = 65.1° [66], which are considerably large. The same DFT protocol was applied to MO2PBN, and the geometrical parameters are available from the experiment, affording antiferromagnetic 2*J*/*k*_B_ = −56.1 K. The experimental value was −73.8 K in the crystalline form [66]. The following known derivative also adds a supportive instance; 5-bromo-2,4-dimethoxy-1,3-phenylene bis(*tert*-butyl nitroxide) (BrMO2PBN, Scheme 3) [67] was reported to have larger torsion (*θ*_1_ = −79.1 and *θ*_2_ = 82.5°). Our DFT calculation suggested 2*J*/*k*_B_ = −109.9 K, while the experiments showed 2*J*/*k*_B_ = −79.3 K. Another sterically crowded biradical, 2,4,6-trimethyl-*m*-phenylene bis(*tert*-butyl nitroxide) (MesBN, Scheme 3), was reported by Rassat et al. [68], and MesBN was separated as *syn*- and *anti*-isomers, each of which was characterized as a ground singlet species. Unfortunately, their crystal structures are unknown. The same DFT protocol was applied to MesBN except that the geometries of the *syn* (*C*_S_) and *anti* (*C*_2_) conformers were computationally optimized at the UB3LYP/6-31G(d) level. The exchange coupling constants were computed as 2*J*/*k*_B_ = −93.6 and −111.4 K, respectively (Figure 5). The observed values are reported to be 2*J*/*k*_B_ = −66 to −86 K by means of electron spin resonance spectroscopy. Further attempts toward novel switching materials using MO2PBN, BrMO2PBN, or MesBN with [Y(hfac)_3_(H_2_O)_2_], based on the same strategy as that of TF2PBN, were aborted in our project since they are already singlet species.

**Figure 6:**
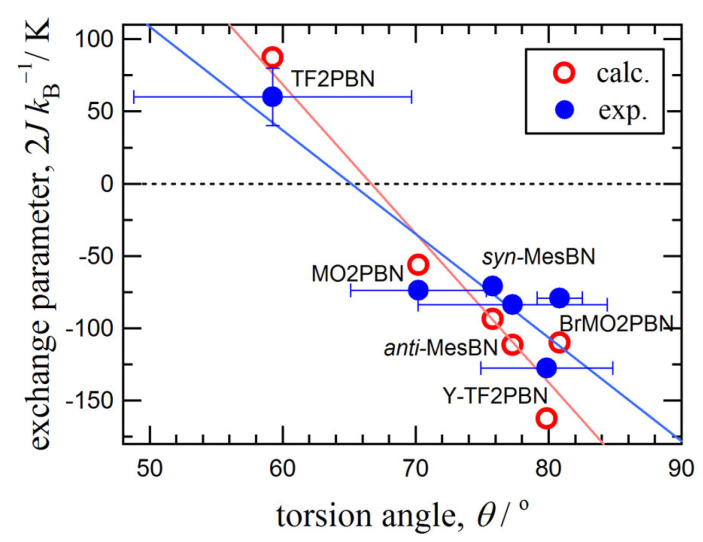
Plot of the exchange coupling constant, 2*J*, vs. the averaged out-of-conjugation torsion angle, *θ* = (|*θ*_1_| + |*θ*_2_|)/2. For the definition of *θ*_1_ and *θ*_2_, see Scheme 2, and the angular error bar implies |*θ*_1_| and |*θ*_2_|. The experimental and calculated 2*J* data are marked in filled and open circles, respectively. The lines represent an empirical linear relationship.

The exchange constant is clarified to be very sensitive to the conformation of the nitroxide group. Now, we can propose a possible magneto-structure relationship (Figure 6). The protecting groups are various (CF_3_, CH_3_, Br, and CH_3_O), but we simplify a model by ignoring such a substituent effect. The exchange coupling constant 2*J* was plotted against the averaged out-of-conjugation torsion angle, *θ* = (|*θ*_1_| + |*θ*_2_|)/2. We can find an almost monotonic negative slope in the observed 2*J*_exp_ vs. torsion angle as well as the calculated 2*J*_calc_ vs. torsion angle. Unexpectedly, electronic substituent effects are hardly observed. The angular dependence of the overlap between the nitrogen 2p_z_ and ipso carbon 2p_z_ orbitals may obey the cos^2^ *θ* law [73,74,75], as expected from the two-fold symmetry of pπ-pπ orbital overlap. Barone et al. reported the computational results from *m*- and *p*-phenylene bridged bisnitroxide models of HNO-C_6_H_4_-NOH [76], and the singlet–triplet energy gap approximately traced cos^2^ *θ*. In magnetic resonance spectroscopy, one may recall the Karplus-Conroy equation [76,77,78,79] and McConnell-Heller equation [11,80], which tell us the NMR and ESR coupling constants, respectively, as functions of the dihedral angle. Here, the angular range is rather small; therefore, an empirical equation is determined via approximate linear fitting. The critical angle, where the sign of 2*J*_exp_ changes from positive (ferromagnetic) to negative (antiferromagnetic), is 65(3)° for the experimental data. For the calculation, the critical angle (66.7(14)°) is very close to the experimental one. The calculation somewhat overestimates the angular sensitivity as a slope. We can verify the literature data on [Mn(hfac)_2_(TF2PBN)_2_] (Table 1). The average *θ* = 63.5° belongs to the border or the ferromagnetic region. The coordination to a divalent cation, Mn^2+^, may cause an electronic state modification of the nitroxide group, for example, the increasing contribution of the canonical formula R^1^–N^+•^(–O^–^)–R^2^ [35], and the magneto-structure picture would be slightly altered for coordination compounds.

We must comment on the role of the H bonds in **1**. The essential function of the H-bonding formation in **1** is to force the π-electron systems to bend more to violate the spin-polarization mechanism or topological rule. A question arises here: why does the *J* value become antiferromagnetic and not approach null? The observable exchange interaction comprises ferro- and antiferromagnetic contributions, namely Equation (4), according to Kahn’s interpretation [81]. The *J*_F_ term originates in the two-electron exchange integral, which is always positive. The *J*_AF_ term is regulated with the overlap and transfer integrals between the two magnetic orbitals, which are negative. According to this model, when the *J*_F_ term is reduced, the *J*_AF_ term becomes decisive. The two terms make comparable contributions in TF2PBN, being responsible for the ground-state switching behavior by means of small structural perturbations like H bonds.

*J* = *J*_F_ + *J*_AF_ (4)

The empirical equation is given as 2*J*_exp_ *k*_B_^−1^/K = 467 − 7.2*θ* (Figure 6). The first and second terms partly imply *J*_F_ and *J*_AF_, respectively. This equation holds for a relatively narrow region of torsional angles, and therefore extrapolation to zero torsion seems to be risky according to a linear or cos^2^ function.

## 5. Conclusions

Steric congestion due to *o*-trifluoromethyl groups seems to decrease ferromagnetic interactions in TF2PBN. A further reduction in ferromagnetic interactions originates in the U-shaped bending deformation owing to attractive H-bonding toward the aqua ligands in the [Y(hfac)_3_(H_2_O)_2_] portion. Here, the DFT calculation provided a reliable explanation and prediction. After comparison of the results among a few known compounds, we found a magneto-structure relationship, namely, the observed 2*J* value vs. the out-of-conjugation torsion angle shows a monotonic negative slope, and the critical torsion angle, at which the sign of 2*J* changes from ferro- to antiferromagnetic, is 65(3)°. The conformational effects on the intramolecular magnetic properties are clearly demonstrated. The ferro- and antiferromagnetic interactions have balanced contributions in TF2PBN and are responsible for the ground-state interconversion between triplet and singlet states by means of small structural perturbations like the presence or absence of H bonds. Since TF2PBN can be recovered from the adduct, a triplet/singlet ground-state switchable material has been successfully realized.

Very recently, H-bonding-assisted molecular design has been proposed for flattening conformation and enhancing ferromagnetic exchange coupling [82]. Combining their strategy and related ideas with the present conclusion, control over coplanar or twisted configurations between aryl and nitroxide groups seems to be feasible, which accelerates the development of novel magnetic-switch-based functional materials.

## Figures and Tables

**Table 1:** Selected bond lengths, angles, and dihedral (torsion) angles for TF2PBN, **1**, and [Mn(hfac)_2_(TF2PBN)_2_].

Compound	TF2PBN ^1^	1	[Mn(hfac)_2_(TF2PBN)_2_]
N1–O1	1.2863(19)	1.2842(16)	1.294(3)
N2–O2	1.2826(13)	1.2850(16)	1.2779(17)
N1–C2	1.423(8)	1.4389(16)	1.421(3)
N2–C6	1.4361(14)	1.4393(16)	1.433(2)
N1–C7	1.505(3)	1.5043(17)	1.512(5)
N2–C10	1.5051(16)	1.5004(17)	1.499(4)
O1–N1–C2	115.80(15)	116.44(11)	115.9(2)
O2–N2–C6	116.58(9)	115.83(11)	115.74(15)
O1–N1–C7	117.8(3)	118.29(11)	117.59(18)
O2–N2–C10	119.81(8)	117.73(11)	119.63(12)
C2–N1–C7	126.36(18)	124.33(11)	126.47(12)
C6–N2–C10	123.58(9)	124.12(11)	123.56(13)
N1–C2–C1	119.55(9)	117.96(12)	118.23(19)
N1–C2–C3	121.0(3)	121.94(12)	121.83(13)
N2–C6–C1	118.85(9)	118.30(12)	118.84(19)
N2–C6–C5	121.4(3)	121.53(11)	121.15(15)
O1–N1–C2–C1	126.69(11) ^2^	101.2262(14)	130.08(16) ^3^
O1–N1–C2–C3	−48.78(14) ^2^	−74.9312(14)	−46.05(19) ^3^
O2–N2–C6–C1	−107.53(14) ^2^	−91.264(2)	−94.9(2) ^3^
O2–N2–C6–C5	69.65(15) ^2^	84.7612(19)	81.4(2) ^3^
O1⋯O2	6.339(16) ^2^	5.8868(1)	6.231(11) ^3^
reference	ref. [36]	this work	Ref. [37]

^1^ Measured at 50 K. ^2^ Calculated in Olex2 [40] from CCDC 182/1278 [36]. ^3^ Calculated in Olex2 [40] from CCDC 151603 [37].

## Data Availability

Data are contained within the article.
